# Dietary Variations in a Multiethnic Parkinson's Disease Cohort and Possible Influences on Nonmotor Aspects: A Cross-Sectional Multicentre Study

**DOI:** 10.1155/2018/7274085

**Published:** 2018-12-18

**Authors:** Anna Sauerbier, Anette Schrag, Pablo Martinez-Martin, Lynsey J. Hall, Miriam Parry, Laurie K. Mischley, Panagiotis Zis, K. Ray Chaudhuri

**Affiliations:** ^1^National Parkinson Foundation International Centre of Excellence, King's College Hospital (KCH) NHS Foundation Trust, London, UK; ^2^Institute of Psychiatry, Psychology & Neuroscience, King's College London, London, UK; ^3^Department of Clinical and Movement Neurosciences, UCL Institute of Neurology, Royal Free Campus, University College London, London, UK; ^4^National Center of Epidemiology and CIBERNED, Carlos III Institute of Health, Madrid, Spain; ^5^Bastyr University Research Institute, 14500 Juanita Dr. NE, Kenmore, WA 98028, USA; ^6^Academic Department of Neurosciences, Sheffield Teaching Hospitals NHS Foundation Trust, Sheffield, UK

## Abstract

Dietary habits may differ between Parkinson's disease (PD) patients of different ethnicities. The primary aim of this cross-sectional analysis was to compare dietary habits in a multiethnic PD population and investigate potential nonmotor differences. All patients completed a dietary habits questionnaire. Besides basic demographics, patients' motor involvement (Hoehn and Yahr (HY)) and nonmotor symptoms (Nonmotor Symptoms Scale; Hospital Anxiety and Depression Scale) were assessed. 139 PD patients were included (mean age 66.8 ± 11.6 years; 61.2% male; mean disease duration 6.2 ± 5.2 years; median HY 3): 47.5% were White, 24.5% Asian, and 28.0% Black African and Caribbean (BAC). We found dietary differences between the groups, including a greater frequency of vegetarians and greater consumption of cumin, turmeric, and cinnamon as well as lower consumption of beef in Asian patients than in White and BAC and greater consumption of chili than in White patients and higher consumption of pork in White than Asian and BAC patients. There were no significant differences in dietary supplement consumption after correction for multiple comparisons. None of the dietary factors examined were associated with differences in nonmotor symptoms. Diet and supplement use vary in PD patients across ethnicities, this is both a problem and opportunity for nutritional medicine research. These data support the importance of considering ethnic diversity as part of recruitment strategy in nutrition and clinical studies.

## 1. Introduction

Parkinson's disease (PD) is a heterogeneous disorder, with considerable phenotypic variability, which may be influenced by genetic, epigenetic, and environmental factors [1]. Amongst the environmental factors, it has been suggested that diet might play a role in PD [2, 3]. For example, it has been reported that patients with PD are less likely than controls to adhere to a Mediterranean diet, consume green tea, coffee, blueberries, avoid dairy products or have higher serum vitamin B6, D, and E levels. Lower adherence to this diet was associated with younger age of onset [3–6]. In addition, higher urate plasma levels in men have been associated with lower risk of PD in a prospective case-control study [7]. Previously, work has also hypothesised that vegan and vegetarian diet might have a beneficial effect on PD [3, 8]. However, to date there is not sufficient evidence to recommend any specific dietary modifications to influence rate and progression of PD [9].

Dietary habits may differ between PD patients of different ethnicities; however, data as to whether this may influence the phenotypic expression of PD are lacking. This study is part of a large multiethnic cohort study of nonmotor aspects of PD and the role of ethnicity (“Nonmotor symptoms of Parkinson's in multi-racial ethnic groups,” United Kingdom clinical research network number 18278). We primarily aimed to compare the dietary habits of a multiethnic PD population using a patient-completed diet questionnaire and address possible links between the different dietary habits and the clinical expression of nonmotor symptoms in patients with PD.

We examined the following concepts:

1. Do dietary patterns differ between PD patients of Black African and Caribbean and Asian groups of either first or second generation resident in the United Kingdom compared to White PD patients [10]?
2. Is dietary supplement use different between the White and the non-White PD groups? Specifically, we would look at intake of vitamin D, turmeric, coffee, and black tea as these have been associated with possible effects on the natural history of PD [2].
3. Is there a separate pattern of phenotypic expression with a focus of nonmotor symptoms of PD with the use of dietary supplements?

## 2. Materials and Methods

### 2.1. Study Design and Study Sites

This was a cross-sectional analysis, and data were collected as part of an ongoing multicentre, prospective, observational real-life study called “Nonmotor symptoms of Parkinson's in multi-racial ethnic groups.” This is an National Institute for Health Research (NIHR) portfolio-adopted study (United Kingdom clinical research network number 18278). Four different sites in South and North London, United Kingdom, were included (King's College Hospital, Lewisham Hospital, Luton and Dunstable Hospital, and Queen Elizabeth Hospital). These areas had preexisting multiethnic PD cohort databases.

### 2.2. Ethical Approval

The study was carried out in accordance with the Declaration of Helsinki and authorised by the local ethics committees. All patients gave written consent prior to taking part in the study procedures.

### 2.3. Patients

All included patients had a confirmed diagnosis of PD as per the United Kingdom Brain Bank criteria [11] and attended the Movement Disorders Clinics in London, United Kingdom, at one of the included sites. In this analysis, we included all patients who had completed a diet questionnaire which was designed for this study and was based on the toolkit developed by Mischley and colleagues [3].

### 2.4. Clinical Assessment

Ethnicity was assessed using the criteria from the Office for National Statistics from the Census 2011 in England and Wales. Besides demographics including age, disease duration, levodopa equivalent daily dose [12], and body mass index (body weight/height^2^), the following clinical assessments were performed:

1. Dietary habits: The patient completed the “diet questionnaire,” a modified version of the food frequency questionnaire (FFQ) used in the “Complementary and alternative medicine in PD (CAM Care in PD) study.” The questionnaire was adapted for the purpose of this study and was simplified and modified based on our experience of patient reports and a literature review of factors relevant to PD. The questionnaire was completed as follows: First, participants were asked if they followed any specific diet in the last 6 months with 12 options (no dietary restrictions, calorie restricted, ketogenic, paleo, low carbohydrate, low fat, low protein, vegan, vegetarian, low salt, low sugar, and other diet). Secondly, participants were asked to tick the box if they had taken any vitamins, supplements, or spices/herbs during the last month. As a next step, participants were asked to give the frequency of the average intake of several foods over the last 6 months. These foods included meat (chicken, beef, pork, and fish), fresh red and nonred vegetables, fresh fruits, cheese, milk, diet and nondiet soft-fizzy drinks, and breads, pasta, grains, soy, and nuts. The questions could be answered by ticking one of the 12 different options ranging from “never” to “4–6 times daily.”
2. Motor assessment included the Hoehn and Yahr (HY) scale [13]. The scale classifies the severity of Parkinsonian symptoms into 5 broad stages and allows disease progression and deterioration to be measured [14]: Stage 1 (unilateral involvement); Stage 2 (bilateral without balance impairment); Stage 3 (bilateral with balance impairment, but physically independent); Stage 4 (unable to walk assisted); and Stage 5 (confined to bed or wheelchair).
3. Nonmotor assessment included the comprehensive nonmotor symptoms scale (NMSS) covering nine different nonmotor symptoms domains (cardiovascular, sleep/fatigue, mood/apathy, perception/hallucinations, attention/memory, gastrointestinal, urinary, sexual, and miscellaneous) [15]. Furthermore, patients completed the Hospital Anxiety and Depression Scale (HADS), a patient-completed scale including 14 different items (7 for depression and 7 for anxiety) [16].

### 2.5. Statistics

Data were stored anonymously in a database and analysed using the Statistical Package for Social Sciences (version 23.0 for Mac; SPSS). Demographics were presented as mean and standard deviation, median and interquartile range and percentage for each variable as appropriate. Normality of distribution was assessed with the Kolmogorov–Smirnov test. As all variables were not normally distributed, nonparametric tests were applied. To investigate if there were statistical differences in categorical variables between the three different ethnic groups (White, Asian, and Black African and Caribbean) Pearson's chi-square test or Fisher's exact test was applied. Furthermore, to calculate if there were statistical differences in continuous not normally distributed variables between the three different ethnic groups (White, Asian, and Black African and Caribbean) the Kruskal–Wallis test was applied. Post hoc analysis was conducted between the different pairs of ethnicities (Asian versus White, Asian versus Black African and Caribbean, and White versus Black African and Caribbean).

For the analysis comparing consumption of a specific dietary item, we divided the groups in “consumption” and “negligible consumption” which was defined as consumption of the dietary item once a month or less than once a month. These were arbitrary cutoffs. The Mann–Whitney *U* test between two groups was applied to investigate differences between these groups.

A *p* value <0.05 was considered to be statistically significant. If applicable, the Bonferroni correction was used to account for multiple comparisons.

## 3. Results

One hundred thirty-nine patients who completed the diet questionnaire were considered for this specific analysis. Among those, 66 (47.5%) were White, 34 (24.5%) Asian, and 39 (28.0%) Black African and Caribbean. Basic characteristics are summarized in Table 1. The three ethnic groups did not significantly differ regarding age, gender, HY, or body mass index. There were differences in disease duration (Asian mean 7.8 ± 7.3 years, Black African and Caribbean mean 4.6 ± 4.2 years, and White population mean 6.3 ± 4.2 years) as well as levodopa equivalent daily doses (mean 763.5 ± 520.7, 536.7 ± 453.4, and 737.5 ± 631.5, respectively).

### 3.1. Types of Diet

The number and frequencies of patients who followed any specific diets (White, Asian, and Black African and Caribbean) are shown in Table 2. We found a statistically significant difference in proportions for patients who followed calorie-restricted, paleo, low-fat, vegetarian, and low-sugar diet (all *p* < 0.05). After correction for multiple comparisons, these differences remained statistically significant only for the vegetarian diet.

Post hoc analysis between the different pairs of ethnicities with a Bonferroni correction showed that Asians followed significantly more frequent a vegetarian diet compared to both groups, White and Black African and Caribbean (26.5% versus 4.5% and 2.6%, respectively) (Table 2).

### 3.2. Use of Supplements between the Three Different Ethnic Groups

The use of supplements is summarized in Table 3 according to each ethnicity. Statistically significant differences were observed in relation to reported intake of vitamin D. However, this difference did not remain statistically significant after correction for multiple comparisons.

### 3.3. Dietary Habits including Herbs and Beverages Such as Coffee, Black, and Green Tea

We found statistically significant differences between the three groups in the consumption of fresh chili, fresh herbs, cumin, turmeric, cinnamon, and green tea. After correction for multiple comparisons, all differences remained statistically significant except for fresh herbs and green tea.

Post hoc analysis between the different pairs of ethnicities with a Bonferroni correction showed that Asian PD patients consumed significantly more frequently cumin (73.5% versus 16.7% and 20.5%), turmeric (70.6% versus 16.7% and 20.5%), and cinnamon (64.7% versus 16.7% and 35.9%) compared to the White and Black African and Caribbean population. Furthermore, the Asian population reported significantly more chili consumption compared to the White which was not significantly different to the Black African and Caribbean PD population (Table 4).

### 3.4. Other Dietary Habits between the Three Different Ethnic Groups

We found statistically significant differences between the three groups in the consumption of beef, pork, fish, cheese, grains, and nuts. After correction for multiple comparisons, only the differences for beef and pork remained statistically significant. Post hoc analysis revealed that White and Black African and Caribbean consumed significantly more beef compared to Asian patients (56.1% versus 48.7% versus 12.1%, respectively), while White consumed significantly more pork compared to the other two ethnic groups (57.6% versus 23.7% versus 9.1%, respectively) (Table 5).

### 3.5. Clinical Parameters and the Effect of Specific Dietary Items

In an exploratory analysis, we compared the nonmotor features in those who reported to follow any specific diet (as listed in Table 2) and consumed specific nutritional supplements (shown in Table 3) and herbs and beverages such as coffee, black, and green tea (shown in Table 4) (irrespective of ethnic background) to those who did not, using the NMSS total score, the nine different NMSS domains, and the HADS total score. Due to the limited sample size, we did not compare the effect of different dietary intakes such as consumption of chicken or beef as shown in Table 5.

After correction for multiple comparisons, we did not find any statistically significant differences in clinical parameters between the groups who consumed any of the dietary items outlined above.

## 4. Discussion

Dietary influences have been proposed as possible modulators for clinical aspects of PD related to ethnicity. To our knowledge, this is one of the first studies to explore the relationship between ethnicity and diet in PD. In spite of a relatively small sample size, we believe that this study provides some interesting observations. Firstly, we examined the dietary profile in patients with PD which may be of relevance to clinical features and confirmed differences in dietary factors between the three different ethnic groups studied (White, Asian, and Black African and Caribbean). As all patients studied in this paper were residents in the United Kingdom, they were exposed to a similar choice of food types. Whilst our findings suggest that the majority of patients with PD in each ethnic group followed no dietary restrictions, there were dietary differences between the multiethnic PD groups: Asian patients with PD, similar to what has been reported in non-PD populations [17], significantly more frequent followed a vegetarian diet and consumed cumin, turmeric, and cinnamon than White or Black African and Caribbean patients. Both Asian and Black African and Caribbean patients consumed less pork than White patients, and Asian patients consumed less beef and more fresh chili than White or Black African and Caribbean patients. Unadjusted comparison also suggested higher green tea consumption in Black African and Caribbean patients than in Asian or White patients, but this difference was no longer significant after adjustment for multiple comparisons. Similar differences in diet have been reported in the overall population of Asian and Black African and Caribbean in the United Kingdom [17].

Secondly, we examined nonmotor features between patients who reported specific diet intake such as vegetarian and intake of specific nutritional supplements and consumption of herbs and beverages such as coffee, black, and green tea (irrespective of ethnic background) and those who did not. This is relevant to address personalized diet, an aspect of the circle of personalized medicine as recently described [18]. We did not find any clinical differences in PD symptoms in relation to these dietary factors. However, given the small number of participants and the number of individuals reporting utilization of specific diets and supplements and herbs, these results should be considered exploratory. In specific cohorts, there is some evidence that some dietary aspects may influence the pattern of Parkinsonism. For instance, in Guadelope, the prolonged intake of fruits of Annonacea has been associated with a high percentage of atypical and nondopamine responsive Parkinsonisms [19]. The Annonacea plant contains isoquinolinic alkaloids and acetogenins, which are neurotoxins, and both are mitochondrial complex I inhibitors resulting in Parkinsonism-type disorders [20]. Similarly, in Guam, the ALS-PDC (amyotrophic lateral sclerosis/parkinsonism-dementia complex) has been largely related to intake of cycad toxin seeds [21]. Cycads contain neurotoxic amino acids *β*-d-glucoside (cycasin) and *β*-*N*-methylamino-l-alanine (BMAA), which are consumed by the Chamorro people in traditional foods, medicines, fruit bat [22, 23].

There have also been reports that dietary intake may influence the rate of manifest established PD: examples include high intakes of fruits, vegetables, whole grains, legumes, poultry, and fish [5]. Further examples are studies reporting an effect of curcumin on PD. Curcumin, derived from turmeric, acts as an anti-inflammatory and antioxidant polyphenol. Research by Liu et al. and Wang et al. established an effect of curcumin on alpha-synuclein-induced cell death, and thioflavins and polyphenols found in fermented black tea also have antioxidising properties [24, 25]. Initial work by Grelle et al. found that, on a cellular level, theaflavins act by preventing and reversing amyloid formation [26]. In addition, it has been suggested that higher urate concentration in the blood could modify the risk of developing PD as well as the progression rate in particular in men [7, 27]. Furthermore, mainly case reports have suggested that vegan and vegetarian diet might be beneficial in PD [8, 28]. The possible underlying mechanism has been suggested to be an improved levodopa absorption and modification of the loss of surviving dopaminergic neurons in the brain [8, 28].

The existence and mechanism of a possible relationship between dietary intake and progression rate of phenotype are unclear, but a link has been postulated [7]. A recent review by Erro et al. alluded on the possible pathomechanisms between nutrition and neurodegeneration including aspects such as altered levels of proinflammatory mediators, microglia activation, and inflammation [9]. In addition, the relationship between neurodegeneration and intestinal microbiota is receiving increasingly attention, which is directly related to dietary habits and PD [29].

This study has several limitations. Firstly, we applied a patient-completed questionnaire, and this may have led to underreporting as patients might underestimate, for instance, the consumption of dairy products [30]. Secondly, the amount of the consumed dietary items was not quantified. Supplement intake was recorded as a binary variable, and we did not assess doses or measure blood vitamin levels in this observational study of self-reported intake. Subtle or dose related difference could therefore not be assessed. The sample size in this study was also comparatively low, and we were therefore not able to conduct statistical analysis on the effect of dietary habits on the clinical expression of PD in specific ethnic groups. However, the results could form the basis of larger prospective population-based studies to further address the questions if there is a possible effect of diet on the clinical symptoms expressionin PD and if this differs between ethnic groups.

## 5. Conclusions

To our knowledge, this is one of the first reports on dietary habits in different ethnic populations living in the United Kingdom affected with PD. Our data suggest that dietary habits differ between different ethnic groups affected with PD. There is conflicting evidence in relation to the impact of diet on PD including the rate of manifest PD, progression rate, and clinical symptoms expression. The identification of strong evidence of the link between diet and PD could have important consequences for the prevention and treatment of this heterogeneous condition. Our results highlight that there are differences in dietary habits between patients with PD from different ethnic groups, and larger, more detailed studies investigating the effect of dietary habits on the clinical expression of motor and nonmotor symptoms are warranted.

## Figures and Tables

**Table 1:** Characteristics in the entire population and subcohorts according to ethnicity.

	Entire population (*N*=139)		White (*N*=66)		Asian (*N*=34)		BAC (*N*=39)		*p* value (between White, Asian, and BAC)
	Mean ± SD	Median (IQR)	Mean ± SD	Median (IQR)	Mean ± SD	Median (IQR)	Mean ± SD	Median (IQR)	
Age (years)	66.8 ± 11.6	67 (58–75)	66.8 ± 12.5	72 (56–76)	66.6 ± 11.9	68 (62–76)	66.9 ± 9.9	66 (58–75)	0.947
Gender, male (%)	61.2		54.5		70.6		64.1		0.268
Disease duration (years)	6.2 ± 5.2	5 (3–9)	6.3 ± 4.2	5 (3–9)	7.8 ± 7.3	5 (3–11)	4.6 ± 4.2	3 (1–7)	0.028
Hoehn and Yahr (median and IQR)	3 (2-3)		3 (2-3)		3 (2-3)		2 (2-3)		0.717
LEDD (mg)	687.5 ± 564.2	580 (350–890)	737.5 ± 631.5	579 (400–883)	763.5 ± 520.7	625 (415–1000)	536.7 ± 453.4	400 (300–700)	0.046
BMI (kg/m^2^)	27.2 ± 5.8	27 (23–30)	27.2 ± 6.0	26 (23–30)	26.0 ± 4.30	27 (23–29)	28.2 ± 6.5	27 (23–31)	0.665

Abbreviations: *N* = number; BAC = Black African and Caribbean; BMI = body mass index; LEDD = levodopa equivalent daily dose; SD = standard deviation; IQR = interquartile range. The Kruskal–Wallis test and Pearson's chi-square test were used to test differences between the three different ethnic groups.

**Table 2:** Dietary habits including specific diets between the three different ethnic groups.

	White (*N*=66)		Asian (*N*=34)		BAC (*N*=39)		*p* value
	*N*	%	*N*	%	*N*	%	
No dietary restriction	50	75.8	20	58.8	23	59.0	0.108
Calorie restricted	4	6.1	0	0	6	15.4	0.034
Ketogenic	0	0	1	2.9	0	0	0.245
Paleo	1	1.5	0	0	5	12.8	0.014
Low carbohydrate	1	1.5	1	2.9	2	5.1	0.689
Low fat	5	7.6	2	5.9	10	25.6	0.018
Low protein	0	0	0	0	1	2.6	0.525
Vegan	0	0	1	2.9	1	2.6	0.274
Vegetarian	3	4.5	9	26.5	1	2.6	0.001^*∗*^^,A,B^
Low salt	4	6.1	3	8.8	8	20.5	0.072
Low sugar	6	9.1	4	11.8	11	28.2	0.025
Other diets	4	6.1	4	11.8	1	2.6	0.318

Abbreviations: *N* = number; BAC = Black African and Caribbean. Fisher's exact test was used to test differences between the three different ethnic groups. ^*∗*^These remain statistically significant after adjustment for multiple comparisons (*p*=0.0042). Post hoc analysis between the different pairs of ethnicities: multiple comparisons were corrected with the Bonferroni method; significant differences are highlighted as A for Asian versus White and B for Asian versus Black African and Caribbean.

**Table 3:** Dietary habits including supplements between the three different ethnic groups.

	White (*N*=66)		Asian (*N*=34)		BAC (*N*=39)		*p* value
	*N*	%	*N*	%	*N*	%	
Vitamin C	10	15.2	6	17.6	4	10.3	0.649
Vitamin D	12	18.2	14	41.2	9	23.1	0.040
Vitamin B6	1	1.5	3	8.8	3	7.7	0.142
Vitamin B12	4	6.1	5	14.7	4	10.3	0.362
Folic acid	1	1.5	1	2.9	4	10.3	0.100
Betaine	0	0	0	0	1	2.6	0.525

Abbreviations: *N* = number; BAC = Black African and Caribbean. Fisher's exact test was used to test differences between the three different ethnic groups. ^*∗*^None remain statistically significant after correction for multiple comparisons (*p*=0.008).

**Table 4:** Dietary habits including herbs and beverages such as coffee, black, and green tea between the three different ethnic groups.

	White (*N*=66)		Asian (*N*=34)		BAC (*N*=39)		*p* value
	*N*	%	*N*	%	*N*	%	
Fresh chili/fresh chili powder	21	31.8	24	70.6	21	53.8	0.001^*∗*^^,A^
Fresh herbs	26	39.4	22	64.7	16	41.0	0.042
Cumin	11	16.7	25	73.5	8	20.5	<0.001^*∗*^^,A,B^
Turmeric	11	16.7	24	70.6	8	20.5	<0.001^*∗*^^,A,B^
Oregano	14	21.2	9	26.5	6	15.4	0.506
Cayenne	6	9.1	9	26.5	8	20.5	0.063
Cinnamon	11	16.7	22	64.7	14	35.9	<0.001^*∗*^^,A,B^
Green tea	8	12.1	8	23.5	15	38.5	0.007
Black tea	31	47.0	14	41.2	16	41.0	0.784
Coffee	43	65.2	16	47.1	20	51.3	0.159

Abbreviations: *N* = number, BAC = Black African and Caribbean. Fisher's exact test was used to test differences between the three different ethnic groups. ^*∗*^These remain statistically significant after adjustment for multiple comparisons (*p*=0.005). Post hoc analysis between the different pairs of ethnicities: multiple comparisons were corrected with the Bonferroni method; significant differences are highlighted as A for Asian versus White and B for Asian versus Black African and Caribbean.

**Table 5:** Other dietary habits between the three different ethnic groups.

	White		Asian		BAC		*p* value
	*N*	%	*N*	%	*N*	%	
Chicken (serving size: 1 large or two small pieces (125 grams))							
Weekly Consumption	58	87.9	23	69.7	32	82.1	0.086
Negligible consumption	8	12.1	10	30.3	7	17.9	

Beef (serving size: 1 large or two small pieces (125 grams))							
Weekly consumption	37	56.1	4	12.1	19	48.7	<0.001^*∗*^^,A,B^
Negligible consumption	29	43.9	29	87.9	20	51.3	

Pork (serving size: 1 large or two small pieces (125 grams))							
Weekly consumption	38	57.6	3	9.1	9	23.7	<0.001^*∗*^^,A,C^
Negligible consumption	28	42.4	30	90.9	29	76.3	

Fish (serving size: 1 large or two small pieces (125 grams))							
Weekly consumption	55	83.3	22	66.7	35	89.7	0.037
Negligible consumption	11	16.7	11	33.3	4	10.3	

Red vegetables (serving size: 1/2 cup)							
Weekly consumption	61	92.4	32	94.1	33	86.8	0.578
Negligible consumption	5	7.6	2	5.9	5	13.2	

Nonred vegetables (serving size: 1/2 cup)							
Weekly consumption	63	95.5	31	93.9	36	92.3	0.894
Negligible consumption	3	4.5	2	6.1	3	7.7	

Fresh fruits (serving size: 1 medium)							
Weekly consumption	65	98.5	31	93.9	38	97.4	0.360
Negligible consumption	1	1.5	2	6.1	1	2.6	

Cheese (serving size: 1 slice, 15 grams)							
Weekly consumption	57	86.4	23	67.6	25	64.1	0.017
Negligible consumption	9	13.6	11	32.4	14	35.9	

Milk (serving size: 1 cup (do not include nondairy milks))							
Weekly consumption	54	81.8	27	79.4	34	89.5	0.468
Negligible consumption	12	18.2	7	20.6	4	10.5	

Diet soft-fizzy drinks (serving size: 1 can)							
Weekly consumption	24	36.4	11	32.4	11	28.2	0.688
Negligible consumption	42	63.6	23	67.6	28	71.8	

Nondiet soft-fizzy drinks (serving size: 1 can)							
Weekly consumption	18	27.3	12	35.3	9	23.1	0.501
Negligible consumption	48	72.7	22	64.7	30	76.9	

Bread (serving size: 1 slice or 1 roll)							
Weekly consumption	63	96.9	32	94.1	37	94.9	0.656
Negligible consumption	2	3.1	2	5.9	2	5.1	

Pasta (serving size: 1 cup)							
Weekly consumption	47	73.4	17	50.0	23	59.0	0.057
Negligible consumption	17	26.6	17	50.0	16	41.0	

Grains (serving size: 1 cup)							
Weekly consumption	54	84.4	33	97.1	39	100.0	0.006
Negligible consumption	10	15.6	1	2.9	0	0.0	

Soy (serving size: 1 cup)							
Weekly consumption	5	7.6	6	17.6	3	7.7	0.290
Negligible consumption	61	92.4	28	82.4	36	92.3	

Nuts (serving size: one serving 1/3 cups)							
Weekly consumption	40	60.6	23	67.6	15	37.5	0.026
Negligible consumption	26	39.4	11	32.4	24	61.5	

Abbreviations: *N* = number; BAC = Black African and Caribbean. Pearson's chi-square test or Fisher's exact test was used to test differences between the three different ethnic groups. ^*∗*^These remain statistically significant after adjustment for multiple comparisons (*p*=0.003). Post hoc analysis between the different pairs of ethnicities: multiple comparisons were corrected with Bonferroni method; significant differences are highlighted as A for Asian versus White, B for Asian versus Black African and Caribbean, and C for White versus Black African and Caribbean.

## Data Availability

The data used to support the findings of this study are available from the corresponding author upon request.
